# Doctors’ practice and attitudes towards red blood cell transfusion at Mthatha Regional Hospital, Eastern Cape, South Africa: A mixed methods study

**DOI:** 10.4102/phcfm.v13i1.2889

**Published:** 2021-06-24

**Authors:** Temitope Adedayo, Don O’Mahony, Olukayode Adeleke, Sikhumbuzo Mabunda

**Affiliations:** 1 Department of Family Medicine and Rural Health, Faculty of Health Sciences, Walter Sisulu University, Mthatha, South Africa; 2 The George Institute for Global Health, Faculty of Medicine and Health, University of New South Wales, Sydney, Australia

**Keywords:** red blood cell transfusion, doctors’ attitudes, doctors’ practice, transfusion thresholds, overtransfusion, descriptive study, qualitative study

## Abstract

**Background:**

Unnecessary blood transfusion exposes recipients to potential harms.

**Aim:**

The aim of this study was to describe blood transfusion practice and explore doctors’ attitudes towards transfusion.

**Setting:**

A hospital providing level 1 and 2 services.

**Methods:**

A mixed-methods study design was used. In the cross-sectional descriptive component, a sample was taken from patients transfused over a 2-month period. Blood use was categorised as for medical anaemia or haemorrhage, and appropriate or not. The qualitative component comprised a purposeful sample for focus group and individual semi-structured interviews.

**Results:**

Of 239 patients sampled, 62% were transfused for medical anaemia and 38% for haemorrhage. In the medical anaemia group, compliance with age-appropriate transfusion thresholds was 69%. In medical anaemia and haemorrhage, 114 (77%) and 85 (93.4%) of recipients had orders for ≥ 2 red blood cell (RBC) units, respectively. In adults ≥ 18 years old with medical anaemia, 47.1% of orders would have resulted in a haemoglobin (Hb) > 8 g/dL. Six doctors participated in focus group and eleven in individual interviews. There was a lack of awareness of institutional transfusion guidelines, disagreement on appropriate RBC transfusion thresholds and comments that more than one RBC unit should always be transfused. Factors informing decisions to transfuse included advice from senior colleagues, relieving symptoms of anaemia and high product costs.

**Conclusion:**

Most orders were for two or more units. In medical anaemia, doctors’ compliance with RBC transfusion thresholds was reasonable; however, almost half of the orders would have resulted in overtransfusion. The attitudes of doctors sampled suggest that their transfusion practice is influenced more by institutional values than formal guidelines.

## Introduction

While blood transfusion is a life-saving procedure that can improve health outcomes, its unnecessary use exposes recipients to potential hazards (e.g. infections and transfusion reactions), an increased risk of death, increases healthcare costs and reduces blood availability.^1^ The most common blood product transfused is red blood cells (RBCs),^2^ and in South Africa, 96% of transfusion recipients receive RBCs.^3^ There is evidence of unnecessary RBC transfusion in studies worldwide with prevalence rates of 46% in the United Kingdom,^4^ 21.4% in Spain^5^ and 23% in Northern Ireland^6^ for adults, and of 55.5% in Uganda^7^ and 66% in South Africa for children.^8^

Current guidelines on RBC transfusion advise transfusion at a haemoglobin (Hb) threshold of ≤ 7 g/dL in adults^9,10^ and at age-appropriate Hb thresholds in children.^11^ In stable adult patients, only one RBC unit should be transfused at a time in most clinical situations (exceptions include acute coronary syndromes, and chronic renal and haematological malignancies),^12,13,14^ and the patient should be reassessed after a one-unit transfusion before considering further transfusion. However, one of the national guidelines suggests a general post-transfusion Hb target of 7 g/dL – 9 g/dL,^15^ and yet, this may require more than one unit to be ordered and administered. Doctors often do not know or use the appropriate transfusion thresholds,^8,16^ and routinely transfuse more than one unit at a time.^17^

Factors influencing doctors’ transfusion behaviour include knowledge and attitudes. Doctors may not know current guidelines^18^ and even if they do, behaviour is influenced by other factors, such as emotion, perceived goals and motivation,^19^ and receptivity to input from colleagues.^20^

There were concerns by the South African National Blood Service over unnecessary transfusion at Mthatha Regional Hospital (MRH). This study was undertaken to describe the transfusion practice at the hospital and explore doctors’ attitudes towards blood transfusion.

## Methods

The study setting was MRH, comprising 298 beds, providing level 1 and 2 services to a socio-economically deprived rural population in the OR Tambo District in the Eastern Cape province.^21^ A mixed-method study design was used. For the quantitative component, a cross-sectional descriptive study was performed. A sample was taken of patients transfused at MRH in the medical, surgical, gynaecology, obstetrics, paediatrics and emergency departments. The desired sample size was calculated using the equation:

$$n=p(1-p)z^{2}/d^{2}$$ [Eqn 1]

for a single proportion (*p*) in a cross-sectional study, where *p* is the anticipated prevalence, d is the desired precision and z is the appropriate value from the normal distribution for the desired confidence.^22^ As the proportion (*p*) of transfused patients was estimated at 0.5 of all blood units ordered, a precision of 0.07 was chosen at the 95% confidence interval (*z* = 1.96). This yielded a minimum sample size (*n*) of 196. In anticipation of missing or erroneous data, 20% was added to this to yield a desired sample size of 236. Consecutive blood transfusion recipients in the months of April and September 2019 were enrolled retrospectively.

Due to the difficulty in evaluating blood loss, all transfusions in patients with acute haemorrhage were considered to be appropriate. Three measures of appropriateness in medical anaemia were calculated. The first measure include compliance with the age-appropriate Hb transfusion thresholds: for neonates in first 24 h and those intubated ≤ 12 g/dL, neonates on continuous positive airways pressure (CPAP) ≤ 11 g/dL, other neonates 7 g/dL – 8 g/dL, all children ≤ 12 years with septicaemia ≤ 10g/dL, non-neonate children without septicaemia ≤ 12 years, children ≥ 12 years < 7 g/dL^23^ and all adults < 7 g/dL.^10^ The MRH transfusion guidelines endorse a threshold of Hb < 7 g/dL for adults. The second measure was the ordering of only one RBC unit at a time. The third measure include compliance with a post-transfusion target of Hb ≤ 8 g/dL in recipients ≥ 18 years old. This target was based on the premise that the maximum Hb threshold for ordering RBC was 6.9 g/dL, and an administration of one unit of blood would raise the Hb by 1 g/dL to 7.9 g/dL.^24^ Due to the difficulty in obtaining valid post-transfusion Hb values, the post-transfusion Hb was estimated based on the number of units ordered and the anticipated rise in patient Hb. An estimated post-transfusion Hb of > 8 g/dL was considered as overtransfusion. One primary diagnosis was assigned by the researchers to each recipient as being the most likely cause of anaemia. Major haemorrhage was defined as bleeding resulting in a systolic blood pressure of < 90 mmHg or a heart rate of > 110 beats per minute in adults.^15^

Data obtained were entered in Microsoft Excel 2016 (Microsoft Corporation, Seattle, WA, United States [US]) and exported to STATA 14.1 (Stata Corp LP, College Station, TX, US) for analysis. Numerical data were explored using the Shapiro–Wilk test for normality and presented using non-parametric statistics (median) and interquartile range (IQR: 75th percentile minus 25th percentile). Numerical variables were compared using the Wilcoxon rank-sum test (Mann–Whitney U test). Categorical variables were presented using frequencies, percentages and graphs, and were compared using the two-sample test of proportions. The level of significance was *p* ≤ 0.05.

For the qualitative component, a purposeful sample of non-specialist doctors (interns, medical officers and registrars) was recruited for individual semi-structured interviews and six non-specialist doctors for a focus group interview. Doctors in these categories can prescribe blood without specialist approval at the study site. For individual interviews, the number of doctors sampled was determined by data saturation, the point at which no new data emerge. The interview guide for individual interviews was a modified version of one used in an Australian study.^16^ Two clinical scenarios were chosen for the focus group discussion. An attitude was defined as ‘an enduring, learned predisposition to behave in a consistent way toward a given class of objects … not as they are but as they are conceived to be’.^25^ Interviews were audiotaped, transcribed and grouped into themes and analysed using content analysis techniques.^26^ Each participant was assigned a code. For example, PI 1, intern, means participant number one individual interview, intern refers to their employment grade and 1 refers to years qualified. PF refers to focus group participants.

### Ethical considerations

Ethical approval was obtained from the Human Research Committee of Walter Sisulu University (Protocol number: 013/2013) and institutional approval from the Eastern Cape Department of Health.

## Results

### Quantitative results

A total of 239 RBC recipients were sampled. All received red cell concentrate.

#### Demography

The age range was 1 day to 91 years. Amongst adults and those ≥ 15 years, there were significantly more female than male participants; however, female participants were younger than the male participants (refer to Table 1 and Table 2). Almost two-thirds of paediatric patients were neonates (*n* = 15, 62.5%), and the median age was 1 month for both male and female participants. Overall, 101 (42%) patients were recorded as HIV-positive and 52 (22%) as HIV-negative. With 30.2% (65/215) of patients transfused, the gynaecology ward had the highest number of non-paediatric recipients transfused. Despite the superior transfusion ratio (2.9:1) for female patients compared with male patients in the medical wards, there was no statistical difference in the proportion of female and male recipients transfused (*p* = 0.181). Male patients were transfused at a significantly lower Hb threshold (median = 5.6 g/dL) than female patients (median = 6.5 g/dL; *p* = 0.039).

**TABLE 1:** Demographics and clinical characteristics of recipients ≥ 15 years (*n* = 215).

Characteristics	Male				Female				*p*-value
	*n*	%	Median	IQR	*n*	%	Median	IQR	
**Sex**	41	19.1	-	-	174	80.9	-	-	< 0.0001
**Age, years**	-	-	39	28	-	-	32	22	0.002
15–29	8	19.5	-	-	66	37.9	-	-	0.0255
30–44	17	41.5	-	-	58	33.3	-	-	0.326
45–59	5	12.2	-	-	22	12.6	-	-	0.938
60+	11	26.8	-	-	28	16.1	-	-	0.108
Hb g/dL	-	-	5.6	2.5	-	-	6.5	3.5	0.039
**HIV status**									
Positive	16	39.0	-	-	76	43.7	-		0.588
Negative	15	36.6	-	-	61	35.1			0.854
Unknown	10	24.4	-		37	21.3	-	-	0.663
**Hospital department**									
Gynaecology	-	-	-	-	65	37.4	-	-	-
Maternity	-	-	-	-	36	20.7	-	-	-
Emergency	13	31.7	-	-	22	12.6	-	-	0.029
Medical	13	31.7	-	-	38	21.8	-	-	0.181
General surgery	15	36.6	-	-	13	7.5	-	-	< 0.0001

Note: Interquartile range = 75th–25th percentile.

Hb, haemoglobin; IQR, interquartile range.

**TABLE 2:** Demographics and clinical characteristics of paediatric recipients (*n* = 24).

Characteristics	Male recipient				Female recipient				*p*-value
	*n*	%	Median	IQR	*n*	%	Median	IQR	
**Sex**	11	45.8	-	-	13	54.2	-	-	0.564
**Age, months**	-	-	1.0	5.9	-	-	1.0	11.6	0.619
< 1	7	63.6	-	-	8	61.5	-	-	0.916
1–12	3	27.3	-	-	4	30.8	-	-	0.851
13–48	1	9.1	-	-	1	7.7	-	-	0.902
Hb g/dL	-	-	7.5	2.8	-	-	8.2	0.4	0.257
**HIV status**									
Positive	7	63.6	-	-	2	15.4	-	-	0.015
Negative	2	18.2	-	-	8	61.5	-	-	0.032
Unknown	2	18.2	-	-	3	23.1	-	-	0.769

Note: Interquartile range = 75th–25th percentile.

Hb, haemoglobin; IQR, interquartile range.

#### Primary diagnosis

**FIGURE 1:**
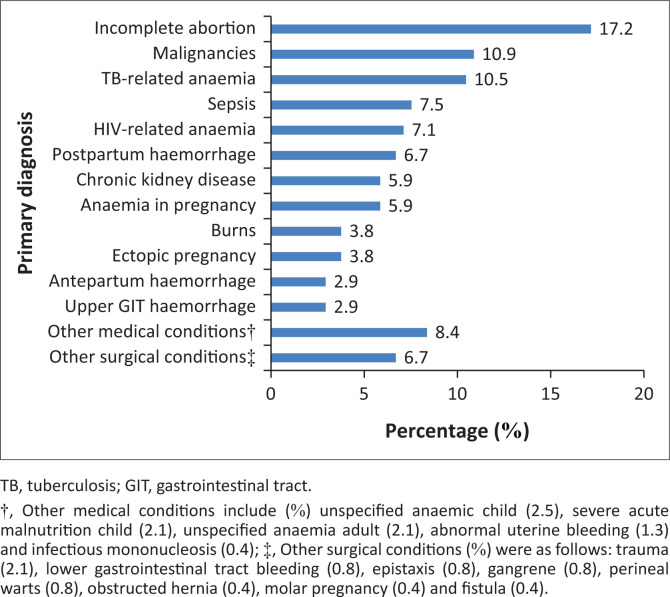
Frequency of primary diagnosis (*N* = 239).

Pregnancy-related conditions constituted 88 (38.8%) of the total diagnoses. The most and least common diagnoses include incomplete abortion and upper gastrointestinal haemorrhage, respectively (see Figure 1).

#### Pre-transfusion haemoglobin

Table 3 shows that those who had a pre-transfusion laboratory Hb < 7 g/dL accounted for 64.5% (*n* = 80) and 52.7% (*n* = 48) of all non-paediatrics patients in medical anaemia and haemorrhage, respectively.

**TABLE 3:** Pre-transfusion haemoglobin in recipients ≥ 15 years (*n* = 215).

Hb ranges (g/dL)	Medical anaemia		Haemorrhage		*p*-value
	*n*	%	*n*	%	
1–2.9	3	2.4	2	2.2	0.915
3–4.9	28	22.6	18	19.8	0.621
5–6.9	49	39.5	28	30.8	0.186
7–8.9	29	23.4	25	27.5	0.495
9–10.9	10	8.1	7	7.7	0.920
≥ 11	5	4.0	11	12.1	0.026

**Total**	**124**	**57.7**	**91**	**42.3**	**0.002**

Hb, haemoglobin.

#### Compliance with transfusion thresholds in medical anaemia

**FIGURE 2:**
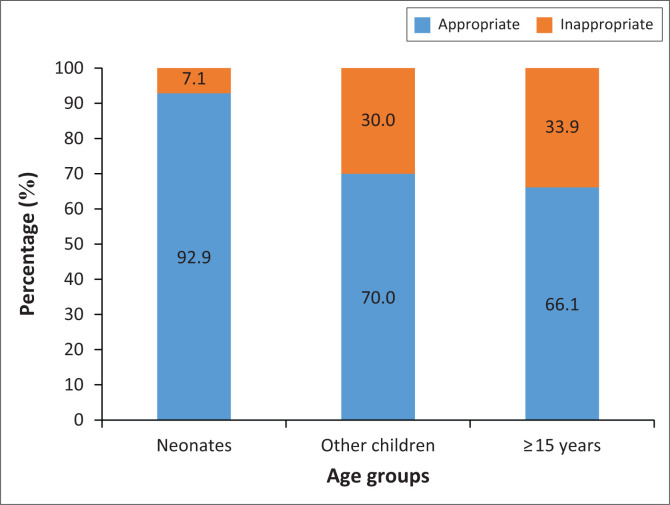
Compliance with the age-appropriate haemoglobin threshold in medical anaemia.

Including all age groups, 69% (102/148) of the recipients were appropriately transfused. Eleven neonates had sepsis, and none required ventilation. In the age group 1–11 months, five children had severe sepsis. All paediatric transfusions were for medical indications (see Figure 2).

#### Red blood cell ordered and transfused

**FIGURE 3:**
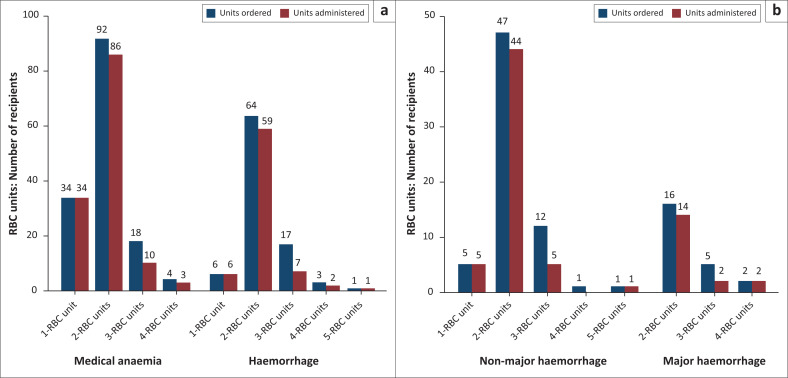
Red blood cell (RBC) units ordered per recipient and units transfused from each order (a & b). Any portion of an RBC unit ordered or administered in paediatric patients was counted as one. Two patients with haemorrhage had no blood pressure or pulse recordings and were excluded from analysis.’

The mode for RBC units ordered and transfused was two in both medical anaemia and haemorrhage, and when one RBC unit was ordered, an equal number was transfused. The only other instance where this was the case is when five RBC units were ordered for a major haemorrhage and all units were transfused. However, when the units were two or more, in all other instances, some of the ordered units were not administered. For instance, 114 (77.0%) of two to four RBC units were ordered for medical anaemia but only 99 (66.9%) were transfused (*p* = 0.052); for haemorrhage 84 (92.3%) of two to four RBC units were ordered but only 68 (74.7%) were transfused, which was statistically significant (*p* = 0.001). There were significantly more one-unit orders (*p* = 0.001) in medical anaemia compared with haemorrhage. For all other orders, there was no significant difference (see Figure 3).

#### Potential overtransfusion

Of RBC orders administered in recipients > 18 years with medical anaemia, it is estimated that 47.1% (58/123) would have resulted in overtransfusion (estimated post-transfusion Hb > 8 g/dL).

### Qualitative results

The age range for individual and focus group participants was 24–45 years; 11 were male participants and six were female participants, comprising nine medical officers, six registrars and two interns. Their years of postgraduate experience ranged between 2 and 15 years. Data saturation was reached after the eleventh individual interview.

#### Use of guidelines and transfusion thresholds

Some participants were not aware of any blood transfusion guidelines:

‘We don’t have an institutional policy on paper regarding blood transfusion.’ (PI 1, intern, 2 years)

‘No guidelines I know of.’ (PI 9, MO, 8 years)

One of the doctors was aware of the aspect of the hospital policy that there should be no transfusion outside normal working hours (except in an emergency):

‘I will transfuse. […] current practice will depend at what time of the day I’m seeing that patient […] because of the guiding laws about transfusion after hours.’ (PF, registrar, 13 years)

Doctors differed in their transfusion thresholds. One of the doctors stated that there was no agreement on the appropriate threshold for transfusion of RBC amongst medical practitioners:

‘We do not have a consensus on the transfusion trigger […] three different doctors can transfuse three different patients. One can transfuse an Hb of 7 [g/dL], another an Hb of 8, another an Hb of 9.’ (PI 6, MO, 4 years)

One of the doctors said that he would not transfuse if the patient was below the transfusion threshold and stable:

‘[*T*]he Hb was 6.6, but clinically the patient is pink and she is not complaining of anything. So that patient you can discharge on haematinics.’ (PI 9, MO, 8 years)

#### Risks and benefits

All doctors interviewed were aware of inherent risks in the administration of blood products. The main risks that they alluded to were the possibility of blood incompatibility and transfusion reactions:

‘For me the most significant risk in blood transfusion is the transmission of diseases in their latent phases. […] But now we also have other risks usually with different types of allergic reactions that could go with the blood transfusion and the severity of which could range from mild to very severe and life threatening.’ (PI 7, registrar, 15 years)

Others mentioned the possibility of infection and fluid overload:

‘Most significant risk for me will be infection. Overload we can … I can deal with that. Incompatibility, that’s fine clearly, I can deal with that.’ (PI 3, registrar, 13 years)

#### Number of red blood cell per order

Some doctors believed the minimum number of RBCs to be transfused is two units:

‘Okay. And I also think that if a patient needs one unit of blood, patient probably doesn’t need blood.’ (PF 6, MO, 3 years)

#### Transfusion based on symptoms

Some doctors considered both the patient’s symptoms and the Hb level before deciding on transfusion:

‘I only transfuse when I really see that there is need to transfuse and the patients are not just having a low Hb but are actually symptomatic.’ (PI 6, MO, 4 years)

#### Cost

All doctors mentioned cost as restricting transfusion behaviour:

‘We also have to be mindful of the cost of blood products. They are very very expensive. And in a public healthcare system where we have to judiciously use our resources [*and*] be very mindful of how much blood you give a patient.’ (PI 8, registrar, 15)

#### Advice from seniors

Two doctors mentioned that their transfusion practice is mainly influenced by advice from senior colleagues:

‘I usually get the opinion from a senior.’ (PI 1, intern, 2 years)

#### Dislike of allogenic blood

One of the doctors felt uncomfortable with the thought of somebody else’s fluids in his body:

‘I am slow to transfuse. I can’t imagine getting somebody else’s fluid in my own body, so I dislike the thought of transfusion. But I guess I have no choice.’ (PI 11, intern, 1 year)

#### Member checks

The qualitative research findings and discussion were emailed to participants for checking in order to ensure accuracy of the data and the researchers’ interpretation. Four responses were received, which stated that the findings were accurate.

## Discussion

The study found that about two-thirds of recipients overall with medical anaemia were appropriately transfused by transfusion threshold. The results of this study in adults are comparable with doctors’ practice in other countries, such as Spain, where 78.6% of adult RBC transfusions in emergency departments were appropriate using thresholds of a Hb < 7 g/dL and Hb < 8 g/dL for acute anaemia and chronic anaemia, respectively,^5^ and in Northern Ireland where 67% of RBC transfusions were appropriate using a threshold of < 7 g/dL for patients under 65 years old and of 8 g/dL for those > 65 years.^6^ Appropriateness in paediatric recipients was better, varying from 93.3% in neonates to 66.7% in the post-neonatal period. In contrast, only 56% of transfusions were inappropriate amongst patients aged 0–5 years at two referral hospitals in Uganda^7^ and while not providing a statistic, Kiguli et al.^27^ also reported that the adherence to World Health Organization transfusion guidelines was poor at hospitals in Kenya, Tanzania and Uganda. While a target of 80% has been used for appropriate transfusion,^28^ it would be apposite for doctors and management at MRH to safeguard patients by agreeing on incremental increases in targets for appropriate transfusion and interventions to achieve them.

Two units were the most common number of RBC units ordered and administered in both medical anaemia and haemorrhage. The MRH guidelines recommend one unit per transfusion in asymptomatic haemodynamically stable adult patients, which is in line with recent guidelines.^12,13,14^ There were more one-unit orders in medical anaemia, suggesting that some doctors follow the guidelines. However, amongst the doctors interviewed, some believed that it was not worthwhile ordering one unit. While one-unit RBC transfusions were discouraged in the past, there has been a paradigm shift in the last decade, and the current best practice is to order and administer one unit as endorsed by the Choosing Wisely campaign ‘[*w*]hy give 2 when 1 will do?’.^29^ This approach also results in cost savings without compromising patient safety.^28,30^

All single-unit orders were administered. Wastage (ordering but not administering) only occurred if more than one RBC unit was ordered at a time (with the exception of one patient who had all five units transfused for a major haemorrhage). Apart from unnecessary costs, wastage may compromise the availability of blood for other patient emergencies.

There was a marked potential overtransfusion (47.1%) in recipients ≥ 18 years old with medical anaemia. Previous studies reported that 19%, 33% and 45% of recipients were overtransfused in Northern Ireland,^6^ UK^17^ and Spain,^5^ respectively. Overtransfusion in these studies was based on a post-transfusion Hb measurement that was > 2 g/dL above the relevant threshold (as opposed to > 1 g/dL in this study). A lower increment was used in this study, as only one RBC unit should be administered at a time to patients with a Hb < 7g/dL,^29^ and one unit would not be expected to raise the Hb above 8g/dL.^24^ Overtransfusion and wastage are also an ethical issue, in that the ethical principle of justice enjoins doctors to avoid wasteful and inefficient practices, so that finite resources are available to others.^31^

There is a disjuncture in guidelines in that some^12,13,14^ advocate one unit be transfused while one national guideline^15^ recommends a post-transfusion Hb target of 7 g/dL – 9 g/dL. This may require transfusion of more than one unit. While the MRH policy recommends one unit per transfusion, more than one unit may be appropriate for patients with a very low Hb.^14,17^ In this study, 25% of recipients ≥ 15 years with medical anaemia had a Hb < 5 g/dL.

The demographic and clinical characteristics of blood transfusion recipients ≥ 15 years old reflect the context of clinical practice at MRH and are similar to those at hospitals in developing countries. The median age of 32 years in females and 39 years in males compares with 33 years for both sexes in a study of blood transfusion practice in Harare.^32^ In recipients aged ≥ 15 years, 81% were females and of these, 76% were in the reproductive age group. Female patients aged 15–29 years had significantly more transfusions than their male counterparts. Pregnancy-related conditions were by far the most common indications for transfusion. These findings are similar to those in a Nigerian tertiary hospital where female participants constituted 69% of all transfused and 75% were in the reproductive age group.^33^ More female participants were transfused at a significantly higher Hb threshold than their male counterparts. It is possible that doctors had a higher threshold for transfusion for pregnancy-related conditions. In developed countries, most RBC transfusions are in older adults (> 60 years) and the predominant indication for RBC transfusion is cardiovascular surgery followed by neoplasms and digestive disorders, and obstetrics and gynaecology constituted 6% of indications.^34^

In this study, paediatric patients aged 0–4 years constituted 10% of recipients, and two-thirds were neonates. There were no children transfused aged between 5 and 15 years; this may be because of children in this age group being preferentially admitted at an adjacent tertiary hospital.

There was a high prevalence of HIV in blood recipients. This is expected in hospitalised patients who have diagnoses related to immune suppression. While anaemia is common in patients with HIV, the indications for transfusion do not differ between patients with or without HIV.^13^

Tuberculosis and HIV with no other comorbidity, comprising 10.5% and 7.1% of diagnoses in recipients, respectively, were important reasons for transfusion in medical anaemia. The two conditions frequently coexist, and amongst patients with HIV in South Africa, the most common reason for severe anaemia is tuberculosis.^35,36^

The study found that some doctors in the qualitative sample did not know or use blood transfusion guidelines, suggesting a challenge in guidelines dissemination and optimal transfusion practice. However, the absence of national South African blood transfusion guidelines may make it difficult for doctors to access agreed standards. Lack of knowledge about institutional transfusion guidelines has also been reported previously in South Africa^18^ and Australia.^16^ Implementation of clinical practice guideline has the potential to change practice and improve outcomes.^37^ There was disagreement on the appropriate RBC transfusion thresholds amongst participants. Similar findings have been reported previously in South Africa^18,38^ and Israel.^20^

The doctors’ decision to transfuse blood was to relieve symptoms attributed to anaemia and not based on the level of Hb alone. The symptoms of anaemia, as detailed by the doctors, are not specific to anaemia.^4^ A review of three studies also has shown no benefit in mortality, morbidity and time to recovery in patients with a Hb level of ≥ 8 g/dL, and transfused because of symptoms of anaemia; although in patients with acute coronary syndrome, there was a trend towards more mortality in the restrictive transfusion group.^39^ Until more definitive evidence is available, it would be advisable that symptomatic patients should only be transfused when symptoms can be attributed to anaemia and not to another pathophysiological process.^4^

Despite a lack of knowledge of guidelines and transfusion thresholds in doctors interviewed, however, in practice, most doctors adhered to the institutional transfusion thresholds for medical anaemia. Participants were knowledgeable about most of the risks of transfusion similar to studies in South Africa^18^ and Australia.^16^ However, none mentioned transfusion-associated circulatory overload (TRACO) or transfusion-related acute lung injury (TRALI) that currently cause the highest number of transfusion associated fatalities,^40^ and can be difficult to recognise and manage.^41^ These findings suggest that doctors interviewed need more training in transfusion-associated reactions.

Doctors’ motivation to limit blood transfusion was mainly influenced by awareness of cost, similar to a Canadian study.^19^ In contrast, some Australian doctors believe that saving life is paramount and blood should be used freely.^16^

One of the doctors mentioned that according to MRH guidelines, no transfusion should be performed outside normal working hours, except during an emergency. This rule may encourage doctors to review their practice and adopt a more restrictive approach to transfusion. Another doctor was uncomfortable with the thought of being infused with another person’s fluid, and this made him consider transfusion carefully.

While doctors did abide by some aspects of current transfusion guidelines, their overall practice was influenced mainly by attitudes that may be ascribed to institutional culture where ‘practice … [is] handed down from one generation of physicians to the next’.^42^

## Limitations and strengths

Limitations of this study include a non-random quantitative sample. However, no difference was observed in the pattern of monthly blood usage, and the risk of selection bias was considered minimal. All patients with haemorrhage were classified as being appropriately transfused irrespective of the Hb threshold. Further research is warranted in this group to assess appropriateness. It is not possible to generalise the qualitative findings. None of the specialists were interviewed. It is important to ascertain their attitudes and practice as they are role models and teachers for non-specialist doctors.

Strengths of this study include extraction of recipient data by a nurse from hospital charts before archiving, thereby minimising the risk of data loss. Method triangulation was ensured using a mixed-method study design to enhance the study’s validity. Triangulation of qualitative data was promoted using individual and group interviews; purposive sampling to ensure rich descriptions of views from doctors with varying years of experience and rank; and member checks to enhance credibility.^43^ It also details doctors’ actual practice of transfusion as opposed to theoretical practice compared with other studies using questionnaires.^18,38^ To the authors’ knowledge, this is the first qualitative study in South Africa on doctors’ attitudes towards blood transfusion.

## Conclusion and recommendations

Doctors’ compliance with RBC transfusion thresholds in medical anaemia was reasonable overall. However, doctors usually ordered more than one unit, contrary to the current best practice, and many orders would have resulted in overtransfusion. The attitudes of the doctors sampled suggest that their transfusion practice is influenced more by institutional values, such as advice from senior colleagues and cost considerations rather than the formal guidelines.

There is an urgent need for evidence-based interventions^28,37^ to improve doctors’ practice of RBC transfusion in patients with medical anaemia in order to ensure that they:

- transfuse at age-appropriate Hb thresholds
- order one unit of blood at a time
- consider and treat underlying conditions that may cause similar symptoms before transfusion based on symptoms of anaemia
- know and use institutional transfusion guidelines.
